# High politics in the Low Countries: COVID‐19 and the politics of strained multi‐level policy cooperation in Belgium and the Netherlands

**DOI:** 10.1002/epa2.1101

**Published:** 2020-11-18

**Authors:** Toon Van Overbeke, Diederik Stadig

**Affiliations:** ^1^ European Institute London School of Economics and Political Science London UK; ^2^ Vrije Universiteit Amsterdam Amsterdam The Netherlands

**Keywords:** COVID‐19, EU, federalism, Low Countries, policy coordination

## Abstract

COVID‐19 presented Europe with an, in many respects, unprecedented challenge. While the virus proved itself to be transnational in nature, not taking heed of borders, government responses were largely national. Still, governments soon found themselves engaged in complex multi‐level policy cooperation at the national, subnational, and supranational levels. This paper looks at the crisis response in the Low Countries (Belgium and the Netherlands) to understand the impact of this process on the political system. We argue that efficient multi‐level policy cooperation in both countries has run up against the limits of existing institutions, leading to significant political grievances. In Belgium, slow negotiation between the central and regional governments has put the federal system in question. In the Netherlands, meanwhile, the absence of European institutions tasked with fiscal policy coordination has increased the salience of the EU fiscal sphere once again.

## 1 INTRODUCTION

The outbreak of the COVID‐19 pandemic in early 2020, in many respects, caught European governments off guard. Not only was the severity of the disease unclear in the early stages of the crisis, but Europe had also “dodged the bullet” during previous health crises, including the swine flu pandemic of 2009–2010 (Versluis et al., 2019), leaving many governments to initially underestimate the risk posed by coronavirus (Capano, 2020).

In this paper, we trace the policy response in Belgium and the Netherlands to COVID‐19. As small and densely populated open economies, the Low Countries^1^ have been among the countries hardest hit by the pandemic in Europe. Between March and September 2020, excess mortality in the Netherlands stood at around 10,203 while Belgium had suffered over 8,000 excess deaths. Unsurprisingly then, the political fallout of the crisis has been substantial in both countries. What is perhaps most surprising, however, is that the public discourse in both countries has not focused on matters of health care to a large degree, but it has rather focused on broader issues of governance, as we shall see. We explain this puzzling lack of focus on health care, amid the largest health crisis the Low Countries have experienced in decades, by examining the problems of multi‐level governance that the COVID‐19 crisis has exposed for these countries. The crisis has laid bare the fact that both countries experience challenges when it comes to finding an optimal allocation and complementarity of tasks between different levels of government. In this regard, we show how the federal system in Belgium caused fatal holdups in decision making throughout the early stages of the pandemic, while the incompleteness of Europe's fiscal institutions allowed negotiations within the Dutch government to become bogged down.

The coronavirus pandemic has presented policymakers with a complex set of challenges. COVID‐19, like similar global health emergencies that went before it, such as the Spanish flu of 1918 and the swine flu pandemic, is a transboundary and multilevel policy problem (Boin et al., 2009; Versluis et al., 2019). That is, the virus does not stop at administrative and regulatory borders and presents policy challenges that require cooperation between governments both horizontally that operate “at different territorial levels” (Bache and Flinders 2004, p3; Thomann et al., 2019). That is, the virus requires the executives of neighboring countries, as well as local, regional, and national governments within any given country to work together. Such cooperation is of course notoriously tricky to establish (Olson, 1982; Putnam, 1988; Scharpf, 2006). We argue that the COVID‐19 crisis has aggravated politically sensitive problems of multi‐level governance, albeit in different ways, in both Belgium and the Netherlands. In the former, the population woke up to the realization that Belgium's federal model with its fragmented policy portfolios had left the country with no fewer than nine ministers for health. As a result, the Belgian crisis response has been riddled with coordination problems which have (re)ignited a debate about the future sustainability of the country's federal model. In the Netherlands, meanwhile, the European Union (EU) has come under major scrutiny in public discourse. Calls for European fiscal solidarity coming from the countries that had been most affected by the pandemic, mostly in southern Europe, paired with a lack of institutionalized resources at the European level to deal with the fiscal aspects of the pandemic, led to an increase in the salience of the EU’s role in fiscal policy in Dutch public discourse. The crisis has therefore sparked debate on the subject of multilevel policy cooperation in both countries, as current institutional arrangements have run into problems of complementarity and regarding the allocation of tasks between different levels of government.

In what follows, we will first present a short overview of the crisis response in the Low Countries. We will then review both countries’ crisis experiences from a comparative perspective. In each case, we will sketch how the health crisis has morphed into a crisis of institutions—of a national nature in the case of Belgium and a transnational one in the case of the Netherlands.

## 2 THE BELGIAN AND DUTCH RESPONSE TO THE CRISIS

Belgium and the Netherlands are both small open economies with a relatively high population density that boast similar continental welfare models (Esping‐Andersen, 1990; Hall & Soskice, 2001). Given these similarities, it should come as no surprise that the Low Countries’ initial responses to the COVID‐19 crisis were strikingly similar in terms of content and timing (as reviewed in section 2.1). As far as economic measures go, both countries broke the bank for a fiscal stimulus to help smoothen the economic hardship of their populations, although Belgium focused more on liquidity guarantees whereas the Dutch approach was more oriented toward direct fiscal stimulus, which we discuss in section 2.2.

### 2.1 Healthcare measures

Belgium and the Netherlands both suffered their first COVID‐19‐related death in the week beginning March 02. The response of both governments, however, had been somewhat lackluster up to that point. Both national governments had stressed that there was no need to cancel national events or to take other measures to prevent the spread of the virus.^2^ Mark Rutte, the Dutch Prime Minister, infamously, shook the hand of the national coordinator of infectious diseases while announcing the Dutch official no handshake policy.^3^

**Figure 1:**
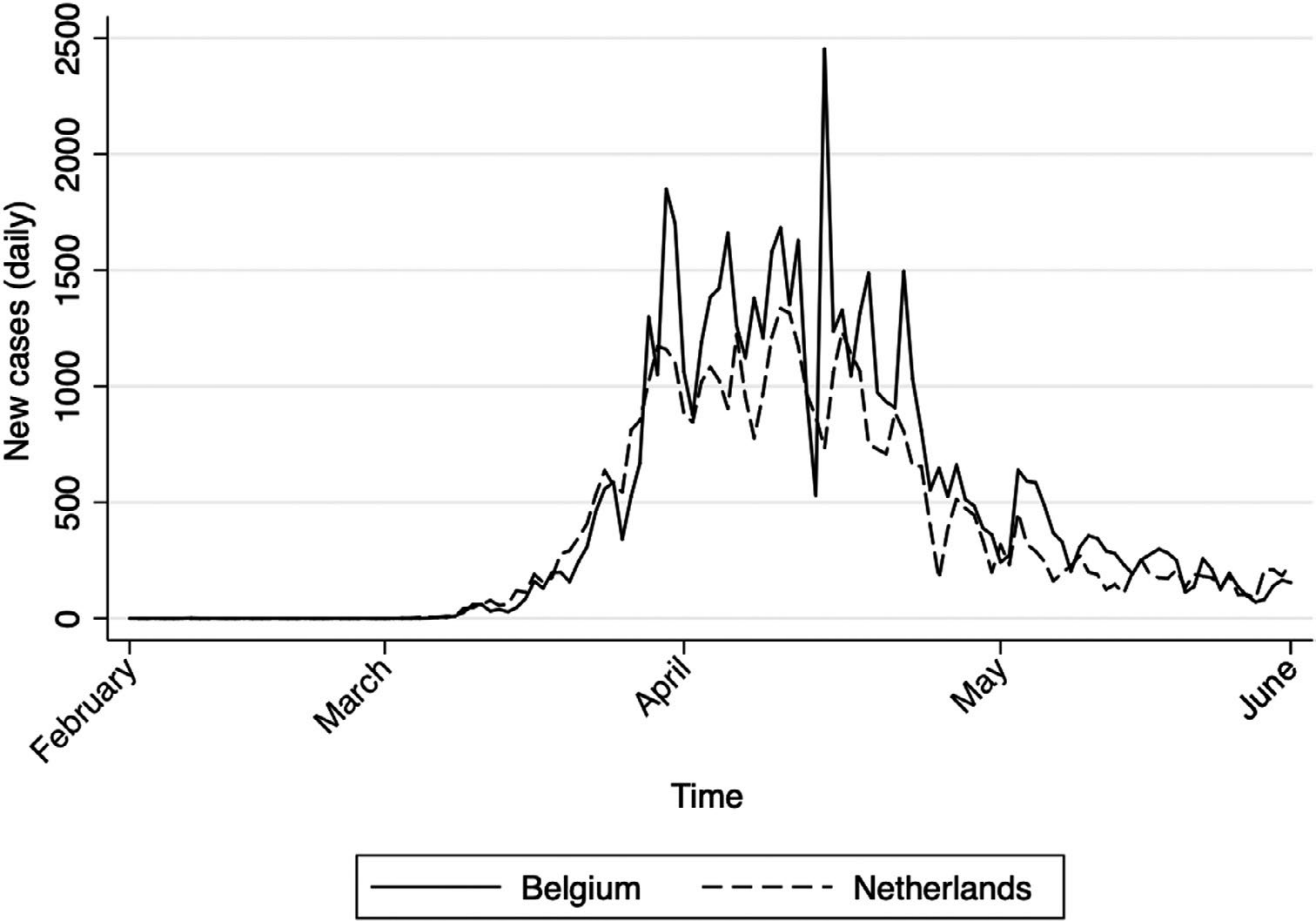
New cases in Belgium and the Netherlands. Source: COVID‐19, Our World in Data (Oxford University)

This wait‐and‐see approach quickly changed once the number of cases accelerated (see Figure 1). Unsurprisingly, the pattern of cases in these neighboring countries is nearly identical. Both countries experienced their first cases in March, followed by an exponential growth of cases from mid‐March to mid‐April, at which point the measures that were introduced started to “flatten the curve” of the virus.

As mentioned previously, the Belgian and Dutch governments followed near‐identical timelines in their response against the disease. Once the seriousness of the pandemic became clear in early March, both governments took unprecedented steps to try to halt the spread of the virus. The Netherlands started by canceling events of more than 100 people and by closing schools, day care facilities, restaurants, bars, and sports facilities on March 12 and March 13. Belgium followed suit by announcing almost identical measures on March 17.^4^ There were only two notable differences in the sanitary responses. Firstly, the Netherlands never banned outside sports and was quick to reopen shops provided a COVID‐19 protocol was adhered to. Belgium, on the other hand, prohibited all non‐essential travel and only reopened non‐essential shops two months later.

Both countries also pursued a similar phased reopening strategy. In the Netherlands schools, daycare facilities, libraries, and other public facilities were reopened on May 11 while Belgium similarly allowed schools and public transport to resume business on the same day.^5^ Over the following weeks, public life in both countries officially restarted as non‐essential travel was again allowed and restaurants, cafes, and gyms started to reopen. In both cases, reopening was subject to strict sanitary protocols (e.g., in Belgium restaurants were obliged to ensure a minimum spacing of 1.5m between tables).

**Figure 2:**
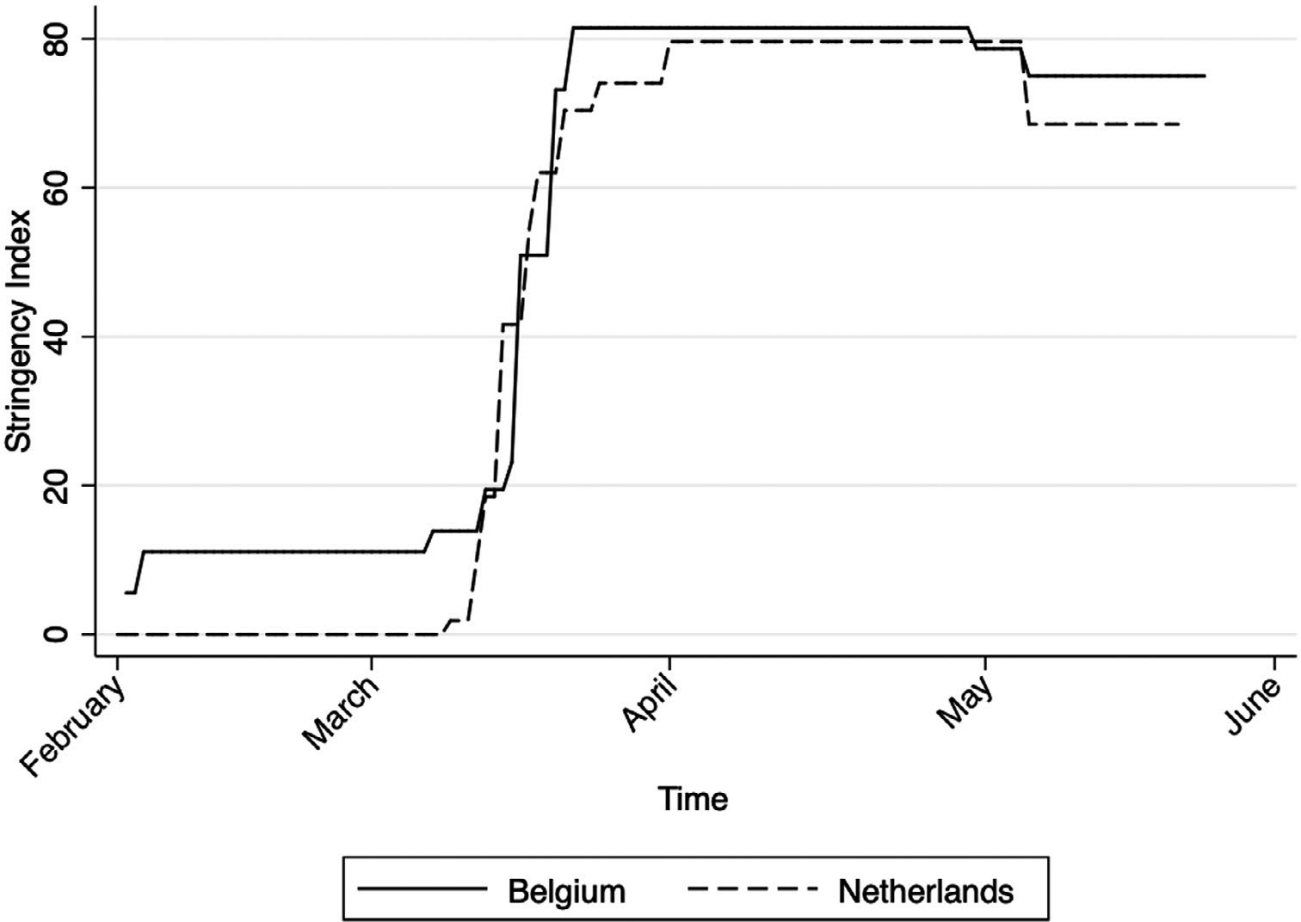
Stringency index of Belgian and Dutch lockdown measures. Source: COVID‐19, Our World in Data (Oxford University)

As described above, Dutch and Belgian healthcare measures were at times mirror images of each other. This is also illustrated by the stringency index (data from the Oxford Blavatnik School) in Figure 2 below. From mid‐March onwards, the two governments put nearly identical measures in place at almost the same time and equally followed a similar phased approach to the reopening of the economy and society from the 11 May onwards.

### 2.2 Economic measures

The fiscal stimulus response enacted by both governments, as was the case with health care, was quite similar, the main difference being that the Netherlands opted for a larger direct fiscal impulse and has allowed more deferrals (such as tax deferrals or grace periods on the repayments of government‐issued loans). Belgium on the other hand focused on liquidity guarantees, as can be seen in Table 1 below.^6^

**Table 1:** Fiscal response to the corona crisis

	Direct fiscal impulse	Deferral	Other liquidity guarantee
Belgium	1.4%	4.8%	21.9%
Netherlands	3.7%	7.9%	3.4%

Source: Bruegel.

In Belgium, the direct fiscal impulse came mostly from the federal level of government; benefits have been increased, temporary unemployment schemes extended, and grants were made available to independent workers. Moreover, VAT was canceled for all restaurants and cafes and all employees were given a €300 cheque to consume in restaurants. The federal Belgian government has also allowed for the deferral of tax payments and business loans, and mortgage loans received a “holiday” of six months. With regard to other liquidity guarantees, the federal government carved out €50 billion for additional credit with a maximum maturity of twelve months guaranteed by banks, with the exception of loan refinancing. Moreover, the federal government has itself started to extend loan guarantees.

The Belgian regional governments have also provided further stimulus. The government of Wallonia pledged a €5,000 one‐off payment to SMEs that had been forced to closed, introduced so‐called “ricochet loans” (with government guarantees and low interest rates aimed at a quick economic restart) for independent workers, and proposed a deferral of loan repayments to government institutions (i.e., with respect to social housing). The package offered by the Flemish government was more comprehensive and included a one‐off payment of €4,000 for SMEs that had been forced to shut and an additional €160 for every day the businesses remained closed after April 04. Moreover, the Flemish government also extended emergency subsidies to heavily affected sectors such as culture and tourism. Additionally, the government, next to a standard loan guarantee scheme, intervened to pay utility bills for those falling into unemployment, and the government also promises tax deferrals (e.g., for road tax) and deferrals to government‐issued loans (e.g., for start‐ups and social housing). Furthermore, the Flemish government created the government‐backed *welvaartsfonds *or welfare fund, which would seek to support Flemish businesses by allowing citizens to invest their savings with a tax break as an incentive. Lastly, Brussels’ regional government also promised emergency funding to the healthcare sector and a one‐off grant of €4,000 for SMEs that had been forced to shut down, €2,000 for those that had to partially close, and a public guarantee for business loans.

The Netherlands announced its first lockdown measures on 17 March. The direct fiscal impulse enacted by the Dutch government in the early months of the crisis amounted to €29.7 billion and primarily consisted of an emergency measure designed to foster employment called *Noodmaatregel Overbrugging voor Werkgelegenheid* or NOW. NOW is €20 billion investment program intended to keep people employed during the crisis. To avoid misuse, companies will receive a 150% fine if they lay off a worker while making use of the scheme—this fine was later reduced to 100%. In addition to NOW, the government handed out a total of €2.45 billion to help independent workers overcome the crisis and a lump sum of €4,000 for businesses that were forced to close down due to government regulations—as had been introduced in the Netherlands. Furthermore, healthcare spending was increased by €800 million and both the agricultural and culture sectors received grants.

Next to the direct fiscal impulse, the Netherlands issued €64 billion in tax deferrals for six months relating to VAT, income tax, wage tax, and corporate tax. Other liquidity measures amount to around €30 billion as additional credit was made available through banks backed by the government. The Dutch government also, somewhat controversially, issued large loans to Dutch multinationals such as KLM and Booking.com which have made the subsidies offered to other heavily affected sectors (i.e., the cultural sector) pale in comparison.

In short, in the early months of the pandemic, both Belgium and the Netherlands pursued an essentially identical lockdown strategy following a highly similar timeline. Moreover, the governments have put together relatively comprehensive economic packages in order to off‐set the high economic costs incurred by the shutdown of social life in the Low Countries. The COVID‐19 crisis has nevertheless exacted a heavy toll in the Low Countries. In terms of excess mortality, Belgium and the Netherlands have suffered over 8,000 and 10,203 deaths, respectively, between March and September 2020. Yet, in spite of suffering a per capita death toll multiple times higher than neighboring countries such as Denmark of Germany, the political fallout of the COVID‐19 crisis in the Low Countries has so far not been dominated by questions of healthcare. In the following sections, we will take a closer look at how the crisis unfolded in Belgium and the Netherlands. We will argue that while institutional overgrow thwarted efficient policy cooperation in Belgium, the absence of such mechanisms at the European level to deal with multilevel fiscal questions has equally opened the door to pollicization of the COVID‐19 crisis in the Netherlands.

## 3 BELGIUM: FEDERALISM’S REVENGE

When COVID‐19 first reared its head in Belgium in late February 2020, the country had been without a governing majority since December 2018. The Michel I cabinet (2014–2018) had been, in part, built on an agreement to postpone dealing with outstanding communitarian issues surrounding the country's federal system. However, this entente had left the Flemish nationalist Nieuw‐Vlaamse Alliantie/New Flemish Alliance (N‐VA)—a constituent part of the governing coalition—exposed on its right flank by the Vlaams Belang/ Flemish Interest party which saw N‐VA opt for an early exit in anticipation of elections in May 2019. Almost a year on, these elections had not yet yielded a majority government. The essential problem in these negotiations was the highly contradictory voting behavior of Flemish and Walloon voters, with the former preferring nationalist right‐wing parties in greater numbers while the center‐left Parti socialiste/Socialist Party (PS) dominates in Wallonia. The crisis did, nevertheless, provide a strong impetus to restart negotiations with the aim of expanding the caretaker government under Prime Minister Sophie Wilmès and to reach a majority for a potential Wilmès II cabinet with full legislative power to tackle the virus.^7^ While several attempts to obtain a majority fell short, a solution was found on March 16 in the form of a temporary minority government with confidence‐and‐supply approach offered by all parties excluding the Flemish nationalist N.VA and Vlaams Belang, but including the hard‐left Parti de Travail de Belgique/ Partij van de Arbeid van België/ Workers’ Party of Belgium (PTB/PVDA).^8^ On top of its expanded legislative power, the newly created Wilmès II cabinet was also given emergency decree powers for a period of six months, allowing the government to circumvent the traditional parliamentary legislative procedures across a range of policy areas and to formulate an effective crisis response. By most standards, this created an extraordinary situation, with a minority cabinet ruling by emergency decree. A deal was therefore struck between parliament and government by which a “super cabinet” would be convened, bringing together all party leaders (excluding the leaders of PTB/PVDA and Vlaams Belang) to take stock of the developing situation on a weekly basis.^9^ Crisis management therefore became an effort that, to an extent, bridged the typically trenchant party divides in Belgium.

While the crisis response has generated some rare political good will at the national level between political rivals in Belgium, attempts at tackling the aforementioned multilevel policy problem have nevertheless run up against the limits of the country's federal system. The first problems, arguably, arose even before the emergence of COVID‐19. The Belgian federal government has traditionally held stocks of personal protective equipment (PPE). Unfortunately, decay meant this stock needed to be destroyed in 2018.^10^ Rather than replenishing these reserves, the then caretaker Wilmès I government decided it would wait before implementing an overhaul of its strategic stock system, preparing a move to a market‐based model. These plans had not yet been brought to a conclusion by the start of 2020, in part due to the legislative limits imposed on the caretaker government. When COVID‐19 hit Belgium, this meant health services quickly ran low on stocks of PPE, leaving the government scrambling to find suppliers on the international markets. Even before the crisis started, therefore, the structural challenges imposed by the federal system on government formation had already complicated Belgium's crisis response.

While government formation has traditionally been a tricky exercise in Belgium, the biggest obstacle to policy response has arguably related to the coordination of policy between the different governments in the federal state. Belgium has a complex structure of interlocking competencies (Deschouwer & Reuchamps, 2013; Swenden & Theo Jans, 2006) involving the federal government and the country's regions and communities.^11^ As a result, as mentioned above, the country boasts nine ministers responsible for different aspects of health policy (or almost 1 for every million inhabitants). Mounting a coherent policy response among these highly interdependent governments was therefore contingent on regular inter‐ministerial conferences and communication that brought together these nine health ministers and other cabinet members with adjacent portfolios.^12^ This setup has proved rigid and has been further hindered by the expansion of the country's case definition—the term used by medical professionals to adjudicate when it is appropriate to test for COVID‐19—partly to make efficient use of scarce testing capacities.^13^ However, both this definition and the decision on which testing facilities would be authorized to carry out tests required inter‐ministerial approval, thereby delaying the expansion of testing capacities even further. While these were highly technical and not particularly salient political issues, this time‐consuming policy process nevertheless allowed the virus to spread relatively unmonitored in the early weeks of the pandemic in Belgium.

Perhaps most revealing of all is the case of Belgium's test‐and‐trace policy.^14^ While a tracing call center was established in cooperation between the regions and the federal government, there remained much confusion about who was responsible for the development of a possible contact‐tracing mobile phone application. This caused significant friction between stakeholders at the inter‐ministerial conferences until the Council of State (Belgium's supreme administrative court) quashed a federal legislative initiative on the matter judging any such application to involve preventative health care, which was the domain of the devolved regional governments.^15^ Continuous devolution over decades has created a maze of interlocking competences in Belgium (Deschouwer & Reuchamps, 2013; Swenden, 2013; Swenden & Theo Jans, 2006). This system has proved both too fragmented to foster an agile policy response to the multilevel health challenge of COVID‐19 and too complex for even the government itself to navigate in times of urgency.

In spite of the significant cross‐party cooperation that can be observed at the outset of the crisis, the virus subsequently reignited the highly contentious debate about the structure and raison d'etre of the Belgian state itself (Reuchamps, 2011; Swenden & Theo Jans, 2006). Belgium's federal minister for health, Maggie De Block, openly acknowledged the fact that the fragmentation of competencies came at the cost of lives, calling the process *“playing Wimbeldon in slow‐motion.”* ^16^ Perhaps most tellingly, then the prime minister herself, Sophie Wilmès, quoting former Prime Minister Yves Leterme, claimed that Belgium's current federal decision making system was *“running on its last fumes.”* In doing so, Wilmès has become the first major Walloon politician to openly call into question the continued existence of the current federal model,^17^ espousing the idea of centralizing key policy domains.

The debate is therefore arguably set up to be more complex than heretofore. Whereas past waves of constitutional reform had generally focused on increased devolution, the shortcomings of Belgium's multilevel response to COVID‐19 is now being seized by parties across the span of Belgian politics to argue both for devolution and for the centralization of competencies. The very architects of the federal system (i.e., the Flemish Christian democrats and the liberals on both sides of the language divide) have started to reconsider the appeal of a stronger federal government. Aside from administrative arguments for such centralization (which are particularly important for the liberals), these new positions are also an attempt to offer a political alternative to the rise of the Flemish nationalist right in recent decades. For these nationalist parties, then, COVID‐19 is seen as an opportunity to push for fully regionalized health care and social security. The Walloon PS party, finally, has been ambiguous in its position regarding constitutional change. Notably, it has opened the door to possible changes, in what has been seen as a trade‐off to secure its socioeconomic priorities in the ongoing government talks. So, in Belgium, the crisis has led to discussions on what the optimal allocation of tasks is between the country's different levels of government, with increased centralization and further devolution on the agenda.

The COVID‐19 crisis, then, has left Belgium in a difficult predicament. The small open economy was struck hard by the health crisis, in spite of pursuing strong lockdown measures. What is more, the political fallout of the emergency has again exposed the structural weaknesses at the heart of the country's existing federal model. Political parties have started to position themselves to move into the inevitable post‐COVID‐19 relaunch of government formations talks. If anything, the re‐emergence of these communitarian issues onto the agenda, as well as the increased salience of socioeconomic policy to tackle the crisis, will further complicate, the already complex, formation talks.

## 4 THE NETHERLANDS: BEING FRUGAL DOESN’T COME CHEAP

In contrast to Belgium, the Netherlands has experienced a period of domestic political stability over recent years. Mark Rutte, the country's current prime minister, has headed three consecutive governments since 2010, the last two of which have not been dissolved prematurely. This is notable given the highly fragmented nature of the Dutch House of Representatives, and the number of crises that have hit during Rutte's tenure, including the financial, euro, refugee, and COVID crises. Each of these episodes actually saw support for Rutte's leadership grow (Bovens & Wille, 2008; Van Lieshout, 2020), whereas support for European fiscal solidarity—which would involve a pooling of common resources at EU level to be distributed centrally, independently of the member states—has decreased. Each crisis listed above also required fiscal cooperation between governments operating at different territorial levels (Bache & Flinders 2004, 3; Thomann et al., 2019). In the absence of an effective mechanism to deal with multilevel policy coordination in fiscal matters at EU level, each crisis would entail lengthy negotiations on emergency measures at the level of the European Council. Multilevel policy coordination in fiscal matters could entail the introduction of a fully fledged fiscal union, which could include a European Minister of Finance or a spending capacity for the European Commission. As discussed by Camous and Claeys (2020), the lack of such institutions only exacerbates existing tensions in the EU. As a traditionally “fiscally hawkish” EU member state, the Netherlands is likely to only continue playing two‐level games (Putnam, 1988), a situation in which the Dutch government would negotiate at the international level by setting the limits of a possible deal in its own parliament, thereby tying its hands in the international negotiations, and further fueling Dutch Euroscepticism.

Since the outset of the COVID‐19 crisis in February, Dutch voters have “rallied around the flag” (Mueller, 1970) and Rutte's popularity has soared. In contrast to the euro crisis, when the Netherlands were a vocal supporter of the EU’s austerity agenda, the response to the current crisis has been surprisingly Keynesian. Minister for Finance Wopke Hoekstra stated that “*the boom, not the slump is the right time for austerity”* ^18^: The Netherlands announced stimulus packages worth around 15% of GDP^19^ and national debt is expected to rise from 48% of GDP before the pandemic to around 75% of GDP in its wake. The focus of the national stimulus package has primarily been on employment and on subsidizing companies in heavily exposed sectors, as was the case in Belgium and throughout much of Europe. This approach and the restrictive measures that were designed to “flatten the curve” of the virus have the approval of an overwhelming majority of the Dutch electorate, with only voters of right‐wing anti‐establishment parties Partij voor de Vrijheid (PVV) and Forum voor Democratie (FvD) tending to disagree with the government's approach to the crisis,^20^ and Rutte's liberal Volkspartij voor Vrijheid en Democratie (VVD) was able to ride on an enormous wave of support throughout the early months of the pandemic.

Meanwhile, the EU has come under major scrutiny in the Netherlands. Calls for European fiscal solidarity from the countries that were most badly affected by the pandemic, mostly in southern Europe, and the lack of institutional resources at EU level to deal with the fiscal aspect of the COVID‐19 crisis, have led to an increase in the salience of the EU fiscal sphere in the Netherlands and to the aforementioned two‐level game (Putnam, 1988).

The cabinet's initial response to calls for a Europe‐wide approach to tackle the pandemic was that the COVID‐19 crisis was not a collective responsibility, but rather one for individual countries, as health care is almost exclusively a national competence. Hence, so argued the Dutch government, EU spending would only be legitimized if it was to compensate for increasing healthcare costs.^21^ Moreover, Minister for Finance Wopke Hoekstra argued that additional financing should always be in the form of loans with conditions for economic reform attached, similar to those loans distributed to crisis‐stricken countries during the euro crisis. Hoekstra's comments were met with outrage by his southern counterparts,^22^ and even former Dutch Finance Minister and Eurogroup President Jeroen “Schnapps und Frauen” Dijsselbloem^23^ argued that Hoekstra's stance was unnecessarily harsh.^24^ However, Hoekstra and Rutte doubled down on their stance while consulting with the Dutch House of Representatives. All parties except the Partij van de Arbeid (PvdA/Dutch Labour Party) and GroenLinks (GL/greens) agreed with the stance of the government, and were opposed to the prospect of European bonds, calling for loans only to be offered with conditions for economic reform attached.^25^ Thus, Hoekstra and Rutte reaffirmed their negotiation position by organizing a vote in parliament, thereby creating a classic two‐level game (Putnam, 1988).

The EU deal that was reached on April 09^26^ was in line with what the Dutch government and parliament wanted, namely a €500 billion stimulus package consisting of European Investment Bank funds and loans from the European Stability Mechanism to be used mostly to cover increasing healthcare costs and unemployment schemes in the member states that had been hit hardest by the pandemic.^27^

Given the government's initial stance and the position of the Dutch parliament, it should come as no surprise that Rutte and Hoekstra were displeased with and outright rejected the plan of Merkel and Macron for a €750 billion European investment that would be partly financed by the issuing of common bonds at EU level.^28^ However, Rutte and the other “frugals” (Austria, Denmark, and Sweden) compromised to support the second EU deal^29^ on July 21, which includes grants and loans, partly financed by jointly issued bonds. Notably, the frugal countries did not support the program before grants were cut from €500 billion to €390 and before budget rebates were reinstated.

The Dutch stance regarding European fiscal issues is not new. Ever since the UK voted to leave the EU in 2016, Rutte has spearheaded the resistance of the so‐called frugal countries against any increases in European spending. This increased the salience of European fiscal policy and, by extension, strengthened the notion that the northern member states, and the Netherlands in particular, have borne disproportionate financial costs for recent European crises. Moreover, the Dutch elections are on the horizon in March 2021. Thus, Rutte does not want his recently built‐up political capital to disappear into thin air, and he is aware that the likes of FvD and PVV will be ready to pounce as soon as Rutte's VVD or other coalition parties adopt a more generous and forgiving stance regarding European fiscal issues.

The current health‐turned‐fiscal crisis has therefore once again aggravated the sentiment in the Netherlands that the European North should not be responsible for southern countries that are sometimes characterized as “fiscally profligate,” which has increased the salience of European fiscal policy in the country's public discourse once again. In the absence of a more effective allocation between national and European levels of government, the Dutch will likely keep playing two‐level games. What's more, the salience of the EU fiscal sphere is likely to keep increasing in the Netherlands with increased levels of Euroscepticism following on its heels if this dynamic persists.

## 5 CONCLUSION

Since it first appeared in Europe in Italy in February 2020, COVID‐19 has hit Europe hard, infecting more than 2.300.000 people and killing an estimated 187.000 between January and June 2020. These staggering figures leave little doubt that COVID‐19 has presented the continent, and indeed the world, with a transboundary problem (Boin et al., 2009) requiring effective multi‐level policy coordination between governments (Versluis, 2019). Such effective cooperation, like any form of collective action, is a tricky exercise (Olson, 1982; Putnam, 1988; Scharpf, 2006; Tsebelis, 2002) in which satisfactory conclusions are often reliant on strong institutions. In this paper, we have compared the crisis response in Belgium and the Netherlands in order to understand why it is that their comparatively severe health crises did not trigger much debate about health policy. We argue that for the Low Countries, the virus has exposed the limits of effective policy coordination for some governance systems.

Belgium and the Netherlands have, in many respects, had a similar crisis experience. Both countries suspended social life in mid‐March, implemented similarly ambitious fiscal packages and reopened their societies in a phased way that followed a comparable timetable. Belgium and the Netherlands have also been regrettably comparable in their public health outcomes as the countries suffered relatively high death tolls. However, in spite of this high cost, the political debate following the COVID crisis has hardly centered around health care as such. Rather, for both countries the post‐COVID political landscape has been shaped by the difficulties encountered by multi‐level policymaking. As Fieldhouse et al. (2020) have argued, crises tend to influence what is on voter's minds. In the Low Countries, COVID‐19 has arguably done exactly this by stirring up already lingering institutional questions as their crisis was marked by the issues and shortcomings of multi‐level policymaking.

Still, despite their similarities, this story has been quite different for each of the Low Countries. In the Netherlands, broad satisfaction with domestic institutions and the crisis response, and impending elections has allowed the incumbent Dutch Prime Minister Mark Rutte to leverage the absence of effective institutions for fiscal coordination in the EU to his own advantage. As we discussed, this Dutch political gamesmanship is a structural and institutional question as opposed to mere coincidence. That is, the absence of solid European fiscal institutions leaves the door open for governments to exploit the need for fiscal cooperation exactly when it is at its greatest. Crisis policymaking in a multilevel context, therefore, requires a minimum level of institutionalization for optimal outcomes to be reached and for unwanted pollicization to be minimized. Belgium, on the other hand, has not even been afforded the luxury to be at odds with Europe. Rather, as the national government's response to the health crisis was thwarted by its complex federal system, the public debate has been dominated by the question of what is next for its strained federal system. In contrast to the case of the Dutch policy on European fiscal redistribution, Belgium shows us that having too many institutions can equally frustrate effective crisis governance. To be sure, this is hardly a new issue on the political agenda in Belgium, but the crisis has clearly highlighted the federal question in the country.

Thus, in light of these cases, it may be asked if regional demands can be married to efficiency? For the Low Countries, COVID‐19 has not just presented itself as an unprecedented health crisis. Rather, debate about the nature of governance has drawn attention away from some of the pertinent questions of health care. In the Low Countries, the virus has exposed the limitations of current institutions and has sparked a debate on the optimal allocation of tasks between different levels of government. Precisely, this lack of effective multi‐level policy cooperation in times of crisis has led to high‐stakes political games, as these cases have shown.

## ENDNOTES

^1^
While the Low Countries is a region in Western Europe bordering the North Sea comprising Netherlands, Belgium, and Luxembourg, it should be noted that this paper focuses only on Belgium and the Netherlands.

^2^

*De Tijd* ( https://www.tijd.be/politiek-economie/belgie/federaal/maggie-de-block-er-is-veel-paniekzaaierij/10211960.html).

^3^
The video went viral on Twitter and can be seen here: https://www.youtube.com/watch?v=pAMsIgzXIjo

^4^

*De Tijd* (https://www.tijd.be/dossiers/coronavirus/strengere-maatregelen-in-belgie-tot-5-april/214892.html).

^5^
Belgian Government, National Security Council. (https://www.belgium.be/nl/nieuws/2020/maatregelen_van_de_nationale_veiligheidsraad_van_24_april_2020).

^6^

*Breugel Thinktank* ( https://www.bruegel.org/publications/datasets/covid-national-dataset/).

^7^

*VRT‐Niewus* (https://www.vrt.be/vrtnws/nl/2020/03/15/onderhandelingen-noodregering-15-maart/).

^8^

*De Morgen* (https://www.demorgen.be/politiek/regering-wilmes-wordt-volwaardig-minderheidskabinet~b1522967/).

^9^

*Knack* (https://www.knack.be/nieuws/belgie/volmachten-dit-is-de-installatie-van-een-voorzittersbewind/article-opinion-1580801.html).

^10^

*Het Laatste Nieuws* (https://www.hln.be/nieuws/binnenland/belgie-vernietigde-stock-uit-2009-van-zowat-zes-miljoen-ffp2-maskers-zonder-ze-te-vervangen~a58fea2c/); *Het Nieuwsblad* ( https://www.nieuwsblad.be/cnt/dmf20200615_04991632); *De Humo* (https://www.humo.be/nieuws/hoe-zit-het-met-de-voorraad-mondmaskers-en-waar-liep-het-mis~b891f4d3/).

^11^

*VRT‐Nieuws* (https://www.vrt.be/vrtnws/nl/2020/05/26/kafka-in-de-gezondheidszorg-wie-zijn-de-9-ministers-en-waarvoor/).

^12^

*Federal Health Ministry of Belgium* (https://www.health.belgium.be/fr/news/conference-interministerielle-sante-publique-du-29-avril-2020).

^13^
In February, to obtain a test, patients were required to be experiencing two out of three recognized COVID‐19 symptoms (i.e., coughing, respiratory problems, and/or fever), needed to have been in contact with someone who had tested positive for the virus, or needed to have returned from a recognised virus hot spot. This definition was later loosened to only require a limited number from a broader set of symptoms.

^14^

*De Tijd* (https://www.tijd.be/politiek-economie/belgie/federaal/het-versnipperde-beleid-heeft-mensenlevens-gekost/10232910.html); *VRT‐Nieuws* (https://www.vrt.be/vrtnws/nl/2020/05/22/hotel-corona/).

^15^

*De Standaard* (https://www.standaard.be/cnt/dmf20200529_04976164); *BusinessSam* (https://businessam.be/het-monster-van-loch-ness-in-de-coronacrisis-beke-wil-dan-toch-een-tracing-app/); *De Standaard* (https://www.standaard.be/cnt/dmf20200602_04978907); *Raad van State* (http://www.raadvst-consetat.be/dbx/adviezen/67424.pdf#search=app); *Knack* (https://trends.knack.be/economie/beleid/philippe-de-backer-over-de-aanpak-van-de-coronacrisis-het-is-een-gigantisch-groepswerk-geweest/article-longread-1603389.html).

^16^

*De Tijd* (https://www.tijd.be/politiek-economie/belgie/federaal/het-versnipperde-beleid-heeft-mensenlevens-gekost/10232910.html).

^17^

*Humo* (https://www.humo.be/nieuws/je-slaapt-niet-gerust-als-er-mensen-sterven-maar-paniek-nooit~bd58db16/).

^18^

*RTL‐Nieuws* (https://www.rtlnieuws.nl/nieuws/politiek/artikel/5120356/minister-van-financien-wopke-hoekstra-komende-tijd-geen).

^19^

*Breugel Thinktank* (https://www.bruegel.org/publications/datasets/covid-national-dataset/#netherlands).

^20^

*European Parliament* (https://www.europarl.europa.eu/at-your-service/files/be-heard/eurobarometer/2020/public_opinion_in_the_eu_in_time_of_coronavirus_crisis/report/en-covid19-survey-report.pdf); *Maurice.nl* (https://www.maurice.nl/peilingen/2020/05/17/corona-crisis-onderzoek-17-mei-2020/).

^21^

*NOS* (https://nos.nl/artikel/2333269-akkoord-over-gebruik-noodfonds-voor-medische-kosten-corona.html).

^22^

*Nos* https://nos.nl/artikel/2328951-hoekstra-te-weinig-empathisch-geweest-schouder-aan-schouder-met-zuid-europa.html

^23^
We are referring here to Dijsselbloem's remarks regarding European fiscal solidarity during the euro crisis. He said to an interviewer of the Frankfurter Algemeine that he cannot spend all his money on liquor and women and expect the interviewer to foot the bill.

^24^

*FAZ* https://www.faz.net/aktuell/wirtschaft/wirtschaftspolitik/nach-interview-in-der-f-a-z-dijsselbloem-ich-bedauere-dass-es-als-nord-gegen-sued-aufgefasst-wurde-14937857.html

^25^

*NRC* https://www.nrc.nl/nieuws/2020/04/07/brede-steun-in-tweede-kamer-voor-opstelling-hoekstra-a3996104

^26^

*European Council* (https://www.consilium.europa.eu/en/press/press-releases/2020/04/09/report-on-the-comprehensive-economic-policy-response-to-the-covid-19-pandemic/).

^27^

*FAZ* https://www.faz.net/aktuell/wirtschaft/wirtschaftspolitik/nach-interview-in-der-f-a-z-dijsselbloem-ich-bedauere-dass-es-als-nord-gegen-sued-aufgefasst-wurde-14937857.html

^28^

*AD* (https://www.ad.nl/politiek/rutte-komt-met-eigen-plan-voor-herstelfonds-hekelt-plan-van-merkel-en-macron~a34bd674/).

^29^

*The Economist* https://www.economist.com/europe/2020/07/21/eu-leaders-agree-on-a-massive-eu750bn-covid-19-recovery-deal
